# Supplementary material for: The Mental Health Burden of Patients with Colorectal Cancer Receiving Care during the COVID-19 Pandemic: Results of the PICO-SM Study
Source: Cancers (Basel). 2023 Feb 15;15(4):1226. doi: 10.3390/cancers15041226 (PMC9954113; doi:10.3390/cancers15041226)
Supplement: Supplementary file 1 [file cancers-15-01226-s001.zip › cancers-2193369-SI.pdf]

Supplementary Table S1

Overview of participants' experience of the COVID-19 pandemic during the study period (2021).

	Number, *n* (%)
**Conditions and/or comorbidities which may increase personal risk of being ill with COVID-19**	
Yes	67 (31.0)
No	125 (57.9)
Prefer not to say	24 (11.1)
**Would like to have testing for COVID-19**, *n* = 158	
Yes	62 (39.2%)
No	96 (60.8%)
**Have had testing for COVID-19**	
**Yes**	**171 (79.2%)**
Tested positive	9/171 (5.3%)
**No**	**43 (19.9%)**
Did not answer	2 (0.9%)
**Have had COVID-19 requiring hospitalisation**	
Yes	3 (1.4%)
No	203 (94.0%)
Did not answer	10 (4.6%)
**Concerned they might get COVID-19**	
Extremely	12 (5.6%)
Very much	18 (8.3%)
Moderately	48 (22.2%)
Slightly	78 (36.1%)
Not at all	54 (25.0%)
Did not answer	6 (2.8%)
**Concerned that COVID-19 had/will have a negative impact on their cancer treatment**	
Yes	58 (26.9%)
No	128 (59.3%)
Don't know	27 (12.5%)
Did not answer	3 (1.4%)
**More concerned about COVID-19 rather than their cancer**	
Yes	14 (6.5%)
No	193 (89.4%)
Did not answer	9 (4.2%)
**Key concerns about cancer treatment and care during COVID-19 pandemic**[a]	
Concerned cancer will come back or progress while waiting for treatment	37 (17.1%)
Where to get help with dealing with side effects	16 (7.4%)
Uncertainty around when treatment or tests will restart	14 (6.5%)
Lack of contact with clinical team	12 (5.6%)
Who to contact if felt cancer has come back or spread	10 (4.6%)
Surveillance scans cancelled and concern over backlog	9 (4.2%)

Supplementary Table S2

Comparison between the demographic characteristics of the longitudinal subgroup of *n* = 95 participants followed-up across two timepoints, 6 months apart. McNemar test or related-samples Wilcoxon signed-rank test were used to compare the participants' answers between timepoints 1 and 2 of the survey.

	**Timepoint 1**	**Timepoint 2**	***P* value**	**Statistical Method**
	**Number, *n* (%)**	**Number, *n* (%)**		
**Gender**				
Male	57 (60.0)	58 (61.1)	0.999	McNemar Test (Male vs Female)
Female	35 (36.8)	35 (36.8)		
Other	1 (1.1)	1 (1.1)		
Prefer not to say	2 (2.1)	1 (1.1)		
**Mean age (years)**	65.0 ± 9.1			
**Ethnicity**				
White/White British	89 (93.7)	90 (94.7)	0.655	Related-Samples Wilcoxon Signed Rank Test
Asian/Asian British (Indian, Pakistani, Bangladeshi)	1 (1.1)	2 (2.1)		
Black/Black British	1 (1.1)			
Other	2 (2.1)	2 (2.1)		
Prefer not to say	2 (2.1)	1 (1.1)		
**Marital Status**				
Single/Divorced/Separated/Widowed	30 (31.6)	31 (32.6)	0.999	McNemar Test
In a relationship/Married/In civil partnership	63 (66.3)	63 (66.3)		
Prefer not to say	2 (2.1)	1 (1.1)		
**Have children**				
Yes	69 (72.6)	71 (74.7)	0.999	McNemar Test (Yes vs No)
No	23 (24.2)	22 (23.2)		
Prefer not to say	3 (3.2)	2 (2.1)		
**Lives alone**				
Yes	23 (24.2)	25 (26.3)	0.999	McNemar Test (Yes vs No)
No	69 (72.6)	69 (72.6)		
Prefer not to say	3 (3.2)	1 (1.1)		
**Previous/Underlying diagnosis of mental health condition**[a]				McNemar Test (Yes vs No)
**Yes**	16 (16.8)	18 (18.9)		
Anxiety	9 (9.5)	8 (8.4)	0.999	
Depression	7 (7.4)	7 (7.4)	0.999	
Panic attacks	1 (1.1)	1 (1.1)	0.999	
Obsessive compulsive disorder	0 (0.0)	0 (0.0)		
Post-traumatic stress disorder	0 (0.0)	0 (0.0)		
Bipolar affective disorder	0 (0.0)	0 (0.0)		
Personality disorder	0 (0.0)	0 (0.0)		
Alcohol/drugs	0 (0.0)	0 (0.0)		
Anorexia	0 (0.0)	0 (0.0)		
Social phobia	0 (0.0)	0 (0.0)		
Other	0 (0.0)	0 (0.0)		
None of the above	78 (82.1)	75 (78.9)	0.804	
Prefer not to say	1 (1.1)	2 (2.1)		

**Self-reported perception of current status of cancer**[a]				
**Stable disease**	66 (69.5)	70 (73.7)		
(Shrinking/responding well to treatment, under control or stable, Have finished treatment & have routine monitoring scans/check-ups, Have had curative treatment & no active cancer)			**0.015**	Related-Samples Wilcoxon Signed Rank Test
**Progressive disease**	7 (7.4)	18 (18.9)		
(Progressing/getting worse)				
**Unknown**	22 (23.2)	7 (7.4)		
(Recently diagnosed & waiting for treatment to start, undergoing investigations at this stage, Don't know or not certain, Other, *Prefer not to say)*				
**Concerned they might get COVID-19**				
Extremely	6 (6.3)	1 (1.1)		Related-Samples Wilcoxon Signed Rank Test
Very much	7 (7.4)	17 (17.9)		
Moderately	20 (21.1)	28 (29.5)	0.065	
Slightly	34 (35.8)	28 (29.5)		
Not at all	26 (27.4)	19 (20.0)		
Did not answer	2 (2.1)	2 (2.1)		
**Conditions and/or comorbidities which may increase personal risk of being ill with COVID-19**			**0.041**	McNemar Test
Yes	27 (28.4)	40 (42.1)		
No	68 (71.6)	55 (57.9)		
**Would like to have testing for COVID-19**,				McNemar Test (Yes vs No)
Yes	25 (26.3)	27 (28.4)	0.581	
No	45 (47.4)	41 (43.2)		
Did not answer	25 (26.3)	27 (28.4)		
**Have had testing for COVID-19**			Test 0.115 Positivity 0.500	McNemar Test (Yes vs No) & (Pos vs Neg)
**Yes**	72 (75.8)	80 (84.2)		
Tested positive	1/72 (1.4)	4/80 (5.0)		
**No**	21 (22.1)	14 (14.7)		
Did not answer	2 (2.1)	1 (1.1)		
**Have had COVID-19 requiring hospitalisation**	1 (1.1)	1 (1.1)		McNemar Test (Yes vs No)
Yes	89 (93.7)	88 (92.6)	0.999	
No	5 (5.3)	6 (6.3)		
Did not answer				
**Concerned that COVID-19 had/will have a negative impact on their cancer treatment**	25 (26.3)	27 (28.4)		Related-Samples Wilcoxon Signed Rank Test
Yes	54 (56.8)	57 (60.0)	0.144	
No	14 (14.7)	9 (9.5)		
Don't know	2 (2.1)	2 (2.1)		
Did not answer				
**More concerned about COVID-19 rather than their cancer**				McNemar Test (Yes vs No)
Yes	8 (8.4)	6 (6.3)	0.727	
No	81 (85.3)	86 (90.5)		
Did not answer	6 (6.3)	3 (3.2)		

**Key concerns about cancer treatment and care during COVID-19 pandemic[a]**				
Concerned cancer will come back or progress while waiting for treatment	17 (17.3)	20 (21.1)	0.804	
Where to get help with dealing with side effects	7 (7.4)	8 (8.4)	0.999	McNemar Test (Yes vs No)
Uncertainty around when treatment or tests will restart	6 (6.3)	10 (10.5)	0.289	
Lack of contact with clinical team	5 (5.3)	10 (10.5)	0.388	
Who to contact if felt cancer has come back or spread	4 (4.2)	9 (9.5)	0.219	
Surveillance scans cancelled and concern over backlog	4 (4.2)	3 (3.2)	0.999	
**Felt COVID-19 pandemic has affected mental health**				
Extremely	1 (1.1)	0 (0.0)		Related-Samples Wilcoxon Signed Rank Test
Very much	3 (3.2)	6 (6.3)		
Moderately	16 (16.8)	14 (14.7)	0.129	
Slightly	21 (22.1)	30 (31.6)		
Not at all	49 (51.6)	43 (45.3)		
Did not answer	5 (3.3)	2 (2.1)		
**Mental health has affected experience of cancer care**				Related-Samples Wilcoxon Signed Rank Test
Yes	7 (7.4)	10 (10.5)	0.868	
No	84 (88.4)	82 (86.3)		
Prefer not to say	4 (4.2)	3 (3.2)		
**Have received support from primary cancer hospital for mental health during COVID-19**				Related-Samples Wilcoxon Signed Rank Test
Yes	6 (6.3)	5 (5.3)	0.235	
No	30 (31.6)	24 (25.3)		
Did not need support	56 (58.9)	59 (62.1)		
Prefer not to say	3 (3.2)	7 (7.4)		
**Wanted more support for mental health during COVID-19**				McNemar Test (Yes vs No)
Yes	5 (5.3)	5 (5.3)	0.999	
No	88 (92.6)	82 (86.2)		
Prefer not to say	2 (2.1)	8 (8.5)		
**Personal coping strategies**				
**Yes[a]**	77 (81.1)	71 (74.7)		
Focussing on positives	60 (63.2)	48 (50.5)	**0.036**	
Using humour	38 (40.0)	33 (34.7)	0.424	
Change in physical activity (e.g. exercise)	33 (34.7)	25 (26.3)	0.152	
Avoiding thinking about it	27 (28.4)	29 (30.5)	0.845	
Planning time	23 (24.2)	14 (14.7)	0.108	
Distracting self	18 (18.9)	17 (17.9)	0.999	McNemar Test (Yes vs No)
Changes in diet (e.g. types of food, amount)	8 (8.4)	12 (12.6)	0.454	
Using religious or spiritual practice(s)	11 (11.6)	14 (14.7)	0.549	
Talking to medical professions	10 (10.5)	7 (7.4)	0.581	
Using meditation, mindfulness or other relaxation techniques	10 (10.5)	12 (12.6)	0.727	

Changing substance intake (e.g. smoking, alcohol, other drugs)	4 (4.2)	4 (4.2)	0.999	
Other	2 (2.1)	2 (2.1)	0.999	
**None of the above**	**18 (18.9)**	23 (24.2)	0.332	
Did not answer	0 (0.0)	1(1.1)		
**Support: Cancer team**				
Extremely	49 (51.6)	49 (51.6)	0.498	Related-Samples Wilcoxon Signed Rank Test
Very much	24 (25.3)	31 (32.6)		
Moderately	6 (6.3)	7 (7.4)		
Slightly	7 (7.4)	5 (5.3)		
Not at all	4 (4.2)	2 (2.1)		
Did not answer	5 (5.3)	1 (1.1)		
**Support: Specialist Nurse**				
Extremely	36 (37.9)	35 (36.8)	0.265	Related-Samples Wilcoxon Signed Rank Test
Very much	17 (17.9)	15 (15.8)		
Moderately	9 (9.5)	15 (15.8)		
Slightly	3 (3.2)	5 (5.3)		
Not at all	14 (14.7)	13 (13.7)		
Did not answer	16 (16.8)	12 (12.7)		
**Support: My GP**				
Extremely	23 (24.2)	15 (15.8)	**0.004**	Related-Samples Wilcoxon Signed Rank Test
Very much	13 (13.7)	13 (13.7)		
Moderately	13 (13.7)	15 (15.8)		
Slightly	9 (9.5)	14 (14.7)		
Not at all	30 (31.6)	34 (35.8)		
Did not answer	7 (7.4)	4 (4.2)		
**Support: Community Services**				
Extremely	20 (21.1)	13 (13.7)	0.093	Related-Samples Wilcoxon Signed Rank Test
Very much	7 (7.4)	15 (15.8)		
Moderately	15 (15.8)	12 (12.6)		
Slightly	12 (12.6)	6 (6.3)		
Not at all	27 (28.4)	38 (40.0)		
Did not answer	14 (14.7)	11 (11.6)		
**Support: Government**				
Extremely	9 (9.5)	8 (8.4)	**0.010**	Related-Samples Wilcoxon Signed Rank Test
Very much	19 (20.0)	8 (8.4)		
Moderately	22 (23.2)	23 (24.2)		
Slightly	14 (14.7)	15 (15.8)		
Not at all	23 (24.2)	34 (35.8)		
Did not answer	8 (8.4)	7 (7.4)		
**Support: Friends/Family**				
Extremely	67 (70.5)	64 (67.4)	0.291	Related-Samples Wilcoxon Signed Rank Test
Very much	18 (18.9)	23 (24.2)		
Moderately	4 (4.2)	2 (2.1)		
Slightly	1 (1.1)	3 (3.2)		
Not at all	1 (1.1)	1 (1.1)		
Did not answer	4 (4.2)	2 (2.1)		

[a]more than one option to question possible

Supplementary Table S3

Univariate and multivariate analyses of factors associated with anxiety (GAD-7 ≥5), depression (PHQ-9 ≥10) and poor well-being (WHO-5 <50), for the longitudinal subgroup of *n* = 95 participants followed-up across two timepoints, 6 months apart. Variables with $P < 0.10$ on univariate analyses were included in the final model respectively. Abbreviations: GAD-7, Generalized Anxiety Disorder scale; PHQ-9, Patient Health Questionnaire-9; WHO-5, World Health Organization Well-being Index. Abbreviations: GAD-7, Generalized Anxiety Disorder scale; PHQ-9, Patient Health Questionnaire-9; WHO-5, World Health Organization Well-being Index.

**Variable**	**Univariate Analysis**			**Multivariate Analysis**		
	**Odds Ratio**	**95% CI**	***P* value**	**Odds Ratio**	**95% CI**	***P* value**
**FACTORS ASSOCIATED WITH ANXIETY (GAD-7 ≥5)**						
Concerned regarding cancer treatment	8.32	2.53-27.38	<0.001			
**Concerned might get COVID-19**	**2.78**	**1.52-5.08**	**0.001**	**4.42**	**1.23-15.88**	**0.023**
**Effect on mental health**	**4.01**	**1.20-13.45**	**0.024**	**2.96**	**1.08-8.14**	**0.036**
Support from Christie	18.13	1.89-174.04	0.007			
Mental health affect care	7.96	1.97-32.19	0.005			
Wanted more support	21.23	2.19-205.51	0.005			
**Past history: Depression**	**12.86**	**2.26-73.02**	**0.004**	**62.03**	**3.87-994.18**	**0.004**
Past history: None of the above	0.22	0.07-0.66	0.005			
Concerned cancer will come back or progress while waiting for treatment	3.88	1.29-11.65	0.012			
Who to contact if felt cancer has come back or spread	3.79	0.91-15.79	0.075			
**Coping: Talking to medical professions**	**6.49**	**1.31-32.04**	**0.028**	**42.42**	**2.47-728.00**	**0.010**
Coping: Avoiding thinking about it	3.33	1.18-9.43	0.019			
**FACTORS ASSOCIATED WITH DEPRESSION (PHQ-9 ≥10)**						
Concerned might get COVID-19	3.69	1.64-8.33	0.002	**18.17**	**2.33-141.92**	**0.006**
Effect on mental health	8.20	2.13-31.56	0.002			
Concerned that COVID-19 had/will have a negative impact on their cancer treatment (Q10)	3.79	0.97-14.79	0.069			
Mental health affect care	19.00	4.18-86.33	<0.001	**51.73**	**1.25-2143.32**	**0.038**
Support from Christie	12.33	1.81-86.33	0.017	**132.09**	**2.16-8064.64**	**0.020**
Wanted more support	42.86	4.19-437.93	0.001			
Past history: Depression	6.42	1.23-33.36	0.043			
Past history: None of the above	0.17	0.05-0.63	0.004			
Concerned cancer will come back or progress while waiting for treatment	3.24	0.90-11.61	0.061			
Where to get help with dealing with side effects	5.20	1.06-25.47	0.027			
Coping: Avoiding thinking about it	3.88	1.12-13.51	0.025			
Support: Specialist nurse	0.66	0.43-1.00	0.051			

FACTORS ASSOCIATED WITH POOR WELL-BEING (WHO-5 <50)						
Past history: None of the above	0.26	0.09-0.79	0.013	**0.11**	**0.02-0.63**	**0.013**
Self-reported perception of current status of cancer Progressive disease/Stable disease	2.79	0.91-8.58	0.067			
Self-reported perception of current status of cancer Unknown/Stable disease	0.73	0.08-6.60	0.999			
Concerned might get COVID-19	2.34	1.35-4.04	0.002	**4.18**	**1.71-10.17**	**0.002**
Effect on mental health	1.84	1.10-3.09	0.021			
Mental health affect care	6.70	1.68-26.73	0.008			
Support from Christie	6.28	0.75-40.77	0.065			
Wanted more support	17.87	1.86-171.51	0.007			
Coping: Focusing on positives	0.31	0.11-0.87	0.023	**0.07**	**0.01-0.38**	**0.002**
Coping: Avoiding thinking about it	3.42	1.15-9.37	0.014	**8.31**	**1.82-38.02**	**0.006**
